# Comparison of conventional MRI and CT scans for assessing bony parameters and classifying On- and Off-Track lesions in anterior shoulder dislocations

**DOI:** 10.1007/s00402-025-06062-w

**Published:** 2025-09-12

**Authors:** Philipp Zehnder, Max Kersten, Markus Schwarz, Peter Biberthaler, Chlodwig Kirchhoff, Lukas Willinger

**Affiliations:** 1 Department of trauma surgery, Technical University of Munich, Munich, Germany; 2 Department of sport orthopedics, Technical University of Munich, Munich, Germany

**Keywords:** Glenohumeral joint, Glenoid morphology, On-/off-track lesion, Shoulder instability, Glenoidal bone loss, MRI/CT

## Abstract

**Background:**

Shoulder dislocation is the most common major joint dislocation, with anterior dislocations accounting for 95% of cases. Accurate assessment of bony lesions, such as glenoid bone loss (GBL) and Hill-Sachs lesions, is critical for treatment planning. While computed tomography (CT) is the gold standard for evaluating bony parameters, magnetic resonance imaging (MRI) may serve as a viable alternative, offering no radiation exposure. This study aims to compare the reliability of conventional 2D (two- Dimensional)-MRI with 2D-CT in measuring bony parameters and classifying lesions as on- or off-track. It was hypothesized that there is no difference in evaluation between MRI and conventional CT scans.

**Methods:**

A retrospective case-control study was conducted on 61 patients (mean age 45 ± 19 years) with anterior shoulder dislocations who underwent both CT and MRI imaging. Radiographic measurements, including glenoid diameter, glenoid defect (in width), Hill-Sachs lesion and bony bridge, were obtained independently from CT and MRI scans. Patients were categorized as on- or off-track based on the glenoid track and Hill-Sachs index. Statistical analyses included correlation tests, Bland-Altman plots, interrater agreement (intraclass correlation coefficient), and sensitivity and specificity analyses for lesion classification.

**Results:**

MRI showed good agreement with CT across most parameters, with mean differences of less than 1 mm for glenoid defect, glenoid diameter, and Hill-Sachs lesions. Correlation coefficients ranged from 0.62 (bony bridge) to 0.93 (glenoid defect). Bland-Altman plots revealed good agreement for glenoid parameters but higher variance for the Hill-Sachs lesion and bony bridge. MRI correctly classified 89% of on-track lesions (sensitivity) and 76% of off-track lesions (specificity). Interrater agreement was excellent for glenoid defect measurements (ICC = 0.962) and lower for the bony bridge (ICC = 0.848).

**Conclusion:**

Conventional MRI demonstrates high reliability in measuring bony parameters and good accuracy in classifying on- and off-track lesions compared to CT. MRI is a viable alternative for preoperative evaluation, particularly in cases with minor bony defects. However, in indeterminate defects, a CT scan is recommended to ensure accurate measurements, classification and treatment planning.

**Level of evidence:**

Level III.

## Introduction

Anterior shoulder dislocation accounts for nearly 95% of all shoulder dislocations with an incidence of 25.2 / 100.000 person-years in the United States [2, 25]. Soft tissue injuries occur in the majority of patients after traumatic anterior shoulder dislocation [11]. Bankart or Perthes lesions are described in up to 97% of patients [9, 22]. For this reason, radiological evaluation using MRI (magnetic resonance imaging) is recommend within the first weeks after anterior shoulder dislocation [24]. The importance of bony stabilizers has increasingly been recognized over the last decade, playing an important role for the treatment success [16]. For example, there is high prevalence of concomitant impression fractures of the posterior humerus (Hill-Sachs lesions) which have been associated with recurrent shoulder dislocations [1]. With the advance in acknowledging the importance of these lesions, the on- and off-track concept was developed [6]. It has become clinical routine to detect these lesions together with the anterior glenoid bone loss (GBL) since both are significant factors for decision making regarding the treatment strategy [7]. Computed tomography (CT) has been established as the gold standard for diagnosing and quantifying concomitant bony injuries [26]. However, it imposes a high dose of radiation for the patient, yet accurate measurement of the bony parameters is crucial [3]. Various studies have shown that 3D MRI is not inferior to CT [18, 23]. It has also been shown that special sequences in MRI, such as ultra-short echo time (UTE) or zero echo time (ZTE), deliver reliable results [8]. However, these methods are not universally available and there is a lack of literature comparing regular multiplanar reconstruction (MPR) between the modalities [4, 5, 8]. Furthermore, there is hardly any literature that verifies whether the measured data ultimately lead to the same classification. The aim of this study was to investigate whether the measured bony parameters can be determined using regular 2D-MRI in comparison to 2D-CT and whether this ultimately leads to a uniform classification of on- and off-track lesions. It was hypothesized that MRI provide a feasible option and their measurements do not differ from CT results.

## Methods

This study has been approved by the ethics committee of the medical faculty of **Technical University Munich (TUM)**, Germany (Project number: 2021-747-S-SR) and was conducted according to the Declaration of Helsinki and their amendments.

This study was designed as a retrospective case-control study. First, all patients who presented with a shoulder dislocation to our level 1 trauma center between 2011 and 2020 were identified. The electronic medical documentation system was used to obtain the demographic data.

Patients with anterior shoulder dislocation and complete CT and MRI imaging of the affected shoulder were included. Exclusion criteria comprised age < 18 years, patients with missing CT or MRI scan, posterior or inferior dislocation. Patients without a Hill-Sachs lesion were not included into the comparison of the classification because the necessary measurements could not be entirely performed and compared.

All radiographic exams (CT and MRI) were performed in the institutions’ radiology department. CT scans were obtained by a 64-slice CT scanner (Somatom Sensation 64; Siemens, Erlangen, Germany) with a slice thickness of 1 mm. MRI was performed by a 3-T MR scanner (Ingenia, Philips, Best, The Netherlands / Ingenia Elition X; Philips Healthcare, The Netherlands). Radiographic parameters were assessed on both CT and MRI scans using a picture achieving and communication system (PACS) work station certified for clinical use (IDS7 21.2, Sectra). Measurements were performed by two raters (orthopedic sports medicine residents with a focus on shoulder and elbow surgery) with experience in musculoskeletal radiography (PZ, MK).

**Fig. 1:**
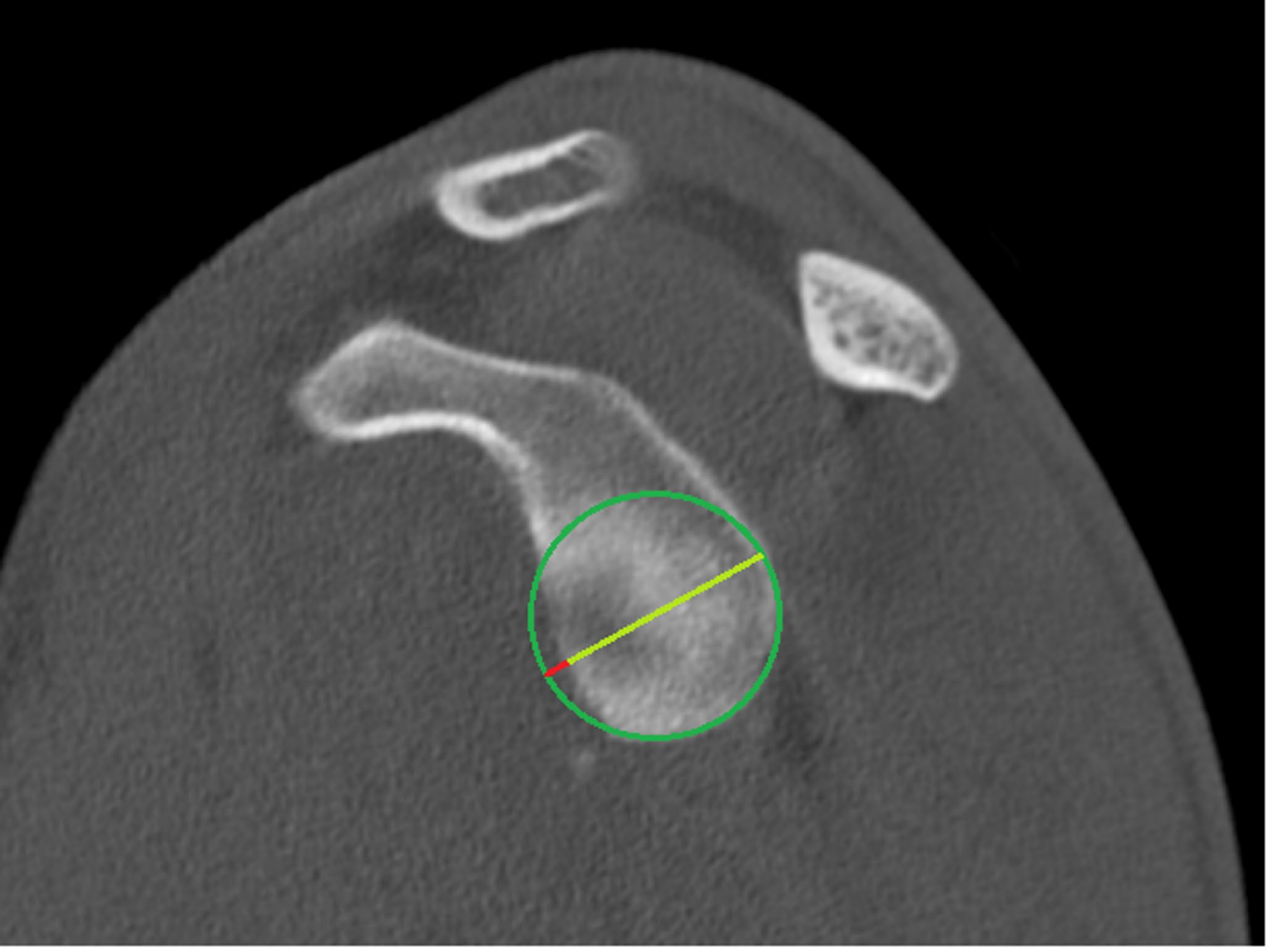
Measurement of the glenoid track using computed tomography as an example. Measurement of the defect length **d (red line)** and measurement of the diameter of the glenoid **D (red line and light green line)**

First, the images were aligned using multiplanar reconstruction. CT scans were assessed and all parameters of interest were obtained. After completion of the CT analysis, the same parameters were measured on MRI at a minimum of four weeks later and in randomized order. The parameters of interest were the diameter of the glenoid (D), the bony defect size of the glenoid (d) and the Hill-Sachs index (Fig. 1).

**Fig. 2:**
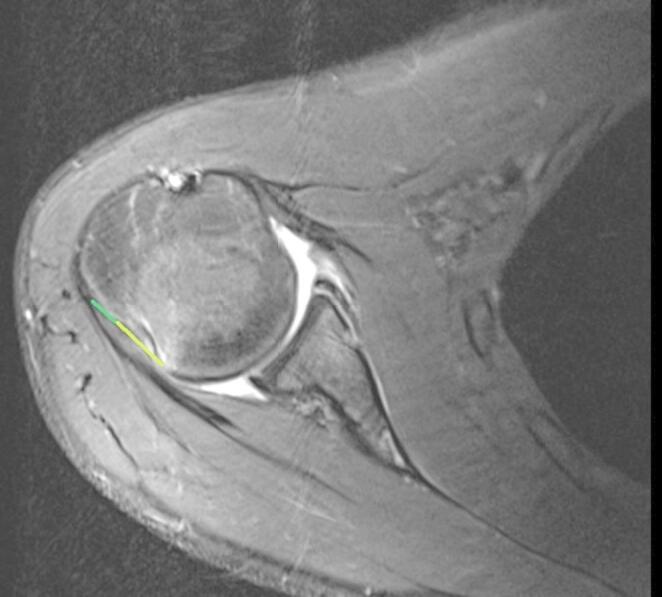
Measurement of the Hill-Sachs Index using MRI as an example. First, measurement of the Hill-Sachs lesion (light green line) and measurement of the bony bridge (green line). Then addition of the two measured distances

The diameter of the lower glenoid was measured with the best-fit-circle method [17]. To calculate the glenoid track the formula (0.83xD - d) was used [6]. The Hill-Sachs Index (HSI) was determined by adding the width of the Hill-Sachs lesion to the width of the bony bridge (representing the intact bone between the rotator cuff insertion and the lateral rim of the Hill-Sachs lesion). Measurements were taken on axial images at the point where the medial extent of the Hill-Sachs lesion was greatest [7] (Fig. 2). If the HSI was larger than the glenoid track, patients were assigned in the off-track group and vice versa [7].

Statistical analysis was conducted using SPSS statistical package software (IBM Corp. Released 2020. IBM SPSS Statistics for Macintosh Version 27.0. Armonk, NY: IBM Corp). The Kolmogorov-Smirnov test was used to assess normal distribution. If normal distribution was confirmed, students’ t-test was used to compare differences in means. Otherwise, Mann-Whitney U test was used for analysis. Categorical variables were compared using the Fisher exact test and the chi-square test (Pearson chi-square). Bland-Altman plots were created to compare the differences between CT and MR measurements. Bland-Altman plots are used to assess the agreement between two quantitative measurement methods by plotting the difference between the methods against their average. They help visualize any systematic bias, random error, or trends in the agreement, and identify whether the differences are clinically acceptable.

Interrater reliability was evaluated by the interclass correlation coefficient (ICC).

*p* < 0.05 was considered statistically significant (Table 3).

**Fig. 3:**
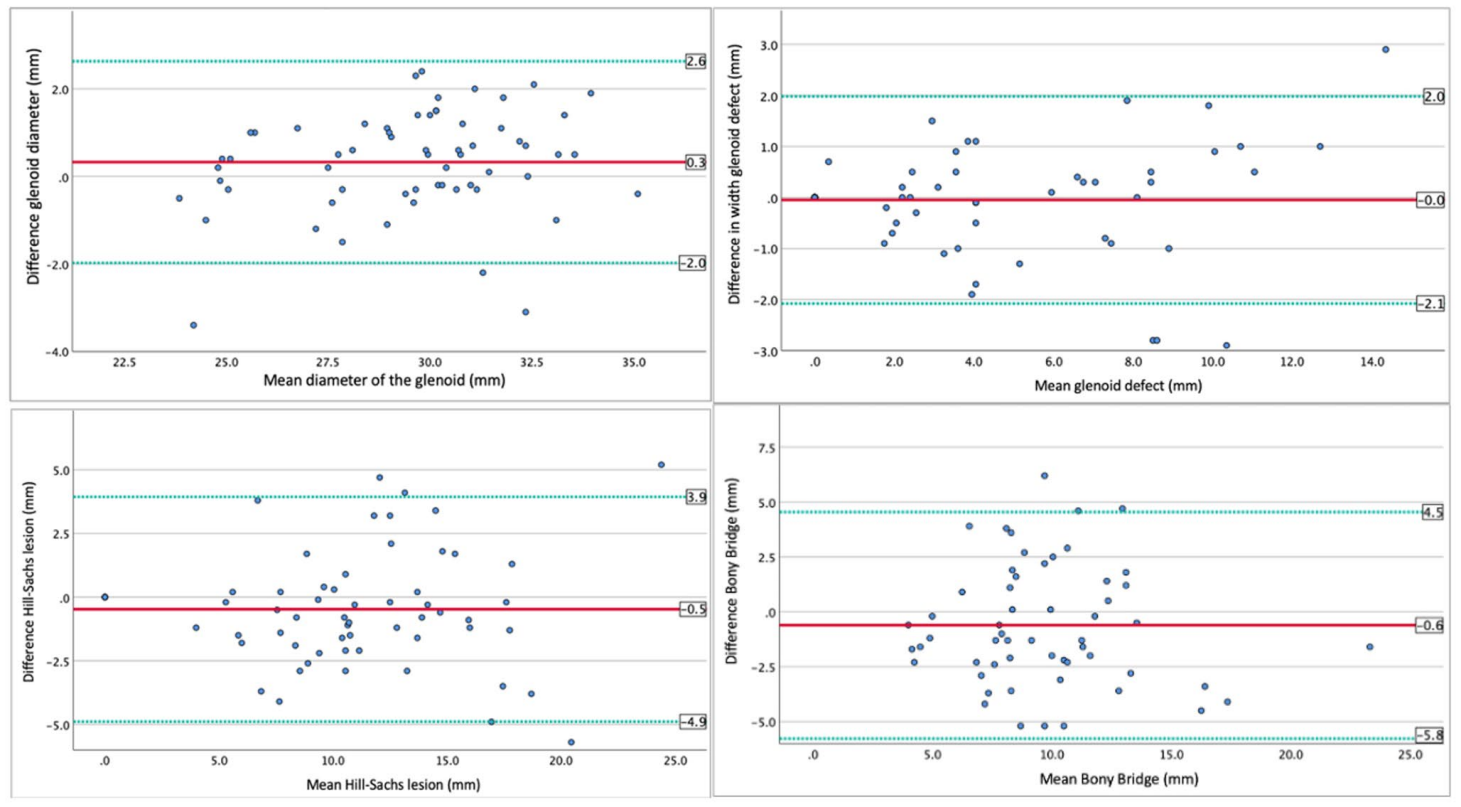
Bland-Altman diagram shows the differences of the MRI compared to the mean values measured in the CT. Limits of agreement 95% (green lines). Average estimation error (red line)

## Results

61 patients (46 men, 15 women) with a mean age of 45 ± 19 years were enrolled in the study. 95% have had a traumatic anterior shoulder dislocation, in the remaining 5% the trauma process was not adequately described or the dislocation was atraumatic. The mean time between CT and MRI was 2.3 ± 18.6 days. Five patients had no measurable humeral bone loss, which is why they were not classified as on-/off track lesions (Table 1).

**Table 1:** Demographics and clinical characteristics of the study population

Total number of patients (*N*)	61
measurable hill-sachs lesion (N)	56
Age (years)	45.3 ± 19.3
Gender (%)	
Male	75.4
Female	24.6
Right	72.1
72.1	72.1
Side (%)	
Left	27.9
Traumatic	95.1
Etiology (%)	
Atrauamtic	4.9
Time between CT and MRI (DAYS)	2.3 ± 18.6

The interrater agreement between the two raters was highest [ICC 0.962 (95% CI 0.943–0.985)] for the measurement of the glenoid defect and lowest [ICC 0.848 (95% CI 0.640–0.933)] for the measurement of the bony bridge.

There was no significant difference between CT and MRI measurements for all assessed radiological parameters.

The measurement of the CT and MRI showed a good to excellent correlation in all parameters (Tables 2 and 3). There was good agreement in the Bland-Altman diagrams for the glenoid diameter and the glenoid defect (95% limits of agreement: diameter − 2.0 to 2.6 mm; defect − 2.1 to 2.0 mm). The limits for the 95% agreement in Bland-Altman diagrams were higher for the Hill-Sachs lesion and the bony bridge (Hill-Sachs: -4.9 to 3.5 mm and bony bridge: -5.8 to 4.5 mm). With regard to the mean estimation error, the MRI overestimated the glenoid size by 0.3 mm, correctly estimated the glenoid defect (± 0.0 mm) and underestimated the size of the Hill-Sachs lesion (-0.9 mm) and the bony bridge (-0.6 mm; Fig. 3). 17 of 19 (89%) on-track lesions on CT scan were correctly identified as on-track lesions on MRI and 28 of 37 (76%) lesions were correctly identified as off-track lesions on MRI (Table 4).

**Table 2:** Students t-test CT/MRI

	Mean Value		Median		Standard Deviation		95% CI		
	CT	MRI	CT	MRI	CT	MRI	CT	MRI	p-value
Diameter of the glenoid (mm)	29.3	29.7	29.7	30.5	2.6	2.9	28.9–30.3	29.2–30.7	0.516
Defect of the glenoid (mm)	4.2	4.1	3.4	3.5	3.8	3.9	3.4–5.4	3.3–5.4	0.946
Hill-Sachs lesion (mm)	11.0	10.5	11.2	10.5	5.2	5.3	10.9–13.1	10.3–12.7	0.619
Bony Bridge (mm)	10.0	9.4	9.3	9.3	3.9	3.7	9.0-11.1	8.4–10.4	0.352

*CI * confidence interval

**Table 3:** Correlation coefficient according to spearman CT/MRT

	ICC	*p*-value
Diameter of the glenoid (mm)	0.89	< 0.001
Defect of the glenoid (mm)	0.93	< 0.001
Hill-Sachs lesion (mm)	0.86	< 0.001
Bony Bridge (mm)	0.62	< 0.001

* ICC* interrater correlation coefficient

**Table 4:** Pearson chi-square classification as On-/Off-track

	CT Off-Track	CT On-Track	total
MRI Off-Track	28	2	30
MRI On-Track	9	17	26
Total	37	19	56

## Discussion

The most important finding of the present study is that conventional MRI can reliably measure bony parameters after anterior shoulder dislocation for determining on- and off-track lesions. MRI showed a high sensitivity and specificity compared to CT scans, demonstrating its potential as an alternative to CT.

It was possible to identify all bipolar bone lesions on both CT and MRI scans. While this has been established in prior studies, our work extended these findings by quantifying the extent of the corresponding bone loss, an area with limited prior exploration [10, 20, 21]. Although several studies described glenoid measurements via 3D-CT or 3D-MRI [13, 18]comparative studies of these modalities in the context of shoulder joint evaluation remain scarce. For instance, Gyftopoulos et al. demonstrated that MRI can measure necessary parameters, comparing these findings with arthroscopy [10]. Similarly, our data, which compared MRI to CT, revealed good to excellent correlation and statistical agreement using both correlation coefficients and comparison of mean values (Tables 2 and 3).

In agreement with Sgroi et al. there was no statistical differences for measuring the Hill-Sachs index [20]. Kimura et al. recently reported that MRI may overestimate the size of Hill-Sachs lesions. While no significant overall difference was observed across the 32 measured cases, the three off-track lesions misclassified on MRI demonstrated significantly overestimated Hill-Sachs lesions [12].

With regard to the size of the Hill-Sachs index, the defects of this study were larger than, for example, those of Gyftiapolus et al. (20.5 mm vs. 16.0 mm). The measured glenoid defect in our study was slightly larger compared to the findings of Gyftopoulos et al. (4.1 mm vs. 2.6 mm). This difference may be attributed to a distinct patient cohort with a higher proportion of off-track lesions (66% vs. 24%)^10^.

Nevertheless, due to the good agreement of the measured values our findings support the usage of MRI in retrospective evaluation of bony parameters after shoulder dislocations.

When classifying lesions as on- or off-track, the presented results support accompany the findings of Gyftopoulos et al. who observed a high sensitivity yet only moderate specificity. To detect an off-track lesion, Gyftopoulos et al. reported a specificity of 72%, compared to 76% in this study. Further, they demonstrated a similar sensitivity of 88% for on-track lesion detection, compared to our 89%.

These findings have significant clinical implications for clinical practice, particularly in guiding imaging modality selection and in preoperative planning for patients with glenohumeral instability. The sensitivity, in particular, warrant further scrutiny. Di Giacomo et al. established a widely accepted treatment protocol based on the on-/off-track concept [6]. Specifically, off-track lesions with substantial glenoid bone loss necessitate bone augmentation of the glenoid (e.g. bone block augmentation), while on-track lesions often receive soft tissue stabilization [7]. However, treating off-track lesions with Bankart repair alone may result in a higher risk of recurrent dislocation [14]. If a bone block augmentation is performed for an on-track lesion, this may be an overtreatment with higher surgical risks for the patient [19].

The presented Bland-Altman diagrams provide valuable insights into why misclassification occurs, in spite of the high comparability of mean measurements. There was an overestimation of the glenoid diameter using MRI, which potentially leads to a larger glenoid track. The overestimation of the glenoid diameter using MRI may be attributed to several factors, including lower spatial resolution compared to CT, partial volume effects, and difficulties in identifying precise bony margins on MRI sequences. At the same time, the Hill-Sachs index was underestimated. This can lead to incorrect classification. Nevertheless, the Bland-Altman diagrams offer a potential clinical decision-making tool. When MRI indicates only minor bony defects within the limits of these diagrams, CT imaging may not be necessary. These results provide clinicians with a preoperative decision-making tool for the possible implementation of CT. It is necessary to optimize the decision-making process in order to achieve the best possible diagnosis and at the same time reduce the economic burden on the healthcare system through the use of additional resources [15]. However, if certain threshold values are exceeded on the MRI findings the presented results indicate preoperative CT scans.

This work has several limitations. It is a retrospective study with its inherited limitations in the selection process of patients and the indication for CT or MRI scans. It is hypothesized that many younger patients underwent MRI without the need for a CT scan, which may account for the older average age of the enrolled cohort. This may be the reason for the high number of off-track lesions. A possible detection of glenoid involvement in the X-ray may have led to an increased indication for CT. CT and MRI scans exhibited differences in slice thickness. However, in clinical reality, the same slice thickness is not always applied and a 3D MRI/CT is by no means distributed over the entire area.

## Conclusion

This study demonstrated that conventional MRI is a reliable alternative to CT for assessing bony parameters (like glenoid diameter, glenoid defect, Hill-Sachs lesion and bony bridge) after anterior shoulder dislocations, with high correlation and agreement between modalities. MRI offers sufficient accuracy for classifying on- and off-track lesions in most cases, while avoiding radiation exposure. However, due to moderate specificity in detecting off-track lesions, CT remains indispensable when MRI measurements approach critical thresholds or when uncertainties arise.

## Data Availability

No datasets were generated or analysed during the current study.
